# Natural Anticancer Molecules and Their Therapeutic Potential

**DOI:** 10.3390/ijms242216066

**Published:** 2023-11-08

**Authors:** Junmin Zhang, Elaine Lai-Han Leung

**Affiliations:** 1 School of Pharmacy, State Key Laboratory of Applied Organic Chemistry, Lanzhou University, Lanzhou 730000, China; 2 Faculty of Health Sciences, MOE Frontiers Science Center for Precision Oncology, University of Macau, Macau SAR 999078, China

Cancer poses a significant global public health challenge [1]. To combat this, the development of targeted anticancer drugs must be ongoing and continuously enriched [2]. An ideal candidate for such drugs would selectively target tumor cells while minimizing harm to normal cells [3]. Specific proteins or enzymes that are highly activated in tumor cell signal transduction pathways are crucial targets for antitumor drug discovery [4]. The discovery of molecularly targeted antitumor drugs that act on these specific targets is key to developing effective antitumor drugs [5]. However, the existing molecularly targeted drug structures are limited, and drug resistance and toxic side effects can be barriers to clinical application. Natural products offer abundant sources and diverse structures, making them ideal for use as hit compounds in structural transformation, modification, and optimization [6,7]. By using natural products with desired biological activities, new chemical entities can be developed that are less prone to drug resistance and toxic side effects. 

This Special Issue aims to showcase the anticancer potential of natural products and their derivatives by isolating, synthesizing, simulating, designing, and conducting clinical trials on these molecules. It is expected to provide a centralized platform for molecularly targeted antitumor drugs that are extracted from natural products and their derivatives, offering a new perspective on the use of natural products in cancer treatment.

We have received several outstanding manuscripts for this Special Issue. Scholars from multiple countries have discovered new anticancer targets and mechanisms within the active molecules of natural products. They have explored active natural products containing peptides based on machine learning/AI technology, as well as popular target inhibitors using the silico method and the impact of chirality on the activity of natural anticancer drugs and derivatives. These achievements reflect the latest progress in the research on natural products as cancer treatments. To aid in the effective understanding of these achievements for readers, we elaborate on them below.

The development of new anticancer drugs involves the identification of the active targets and mechanisms of action of natural products [8]. Curcumin and plumbagin are two such natural products that have multiple targets and significant anticancer activity [9,10]. Though both compounds have been reported to target the PI3K/Akt/mTOR pathway [11,12], the combined effects of these two compounds on cancer cells are still unknown. A study by Tabrz et al. reveals the synergistic effect of curcumin and plumbagin on cancer cells and the possible dose advantage of using them in combination [13]. The study uses molecular docking and molecular dynamics (MD) analysis to investigate the synergistic effect of the two compounds on the PI3K/Akt/mTOR pathway. These findings pave the way for further research on the effects of combination therapy on cancer cells in both in vitro and in vivo models.

MEK2 is a popular target for the development of new anticancer drugs [14]. Many MEK1/2 inhibitors have been approved and widely used as anticancer drugs. Rehan et al. have discovered new MEK2 inhibitors from flavonoids using virtual screening, molecular docking analysis, pharmacokinetic prediction, and MD simulation methods [15]. Their proposed flavonoid compounds are potential inhibitors of MEK2 and are candidate drugs for cancer treatment. 

Tubeimoside-1 (TBMS-1) is an anticancer molecule derived from the Chinese medicinal herb *Bolbostemma paniculatum* (maxim) franquet [16]. Kwon et al. report the sensitization effect of TBMS-1 on the tumor necrosis factor-related apoptosis-inducing ligand (TRAIL) in cancer cells [17]. Their research results indicate that the combination of TBMS-1 and TRAIL therapy increases tumor cell apoptosis. Further mechanism studies have shown that TBMS-1 significantly increases sensitivity to TRAIL by downregulating the ubiquitinase STAMBPL1 (DUB) to disrupt c-FLIP expression. Importantly, the combination of TBMS-1 and TRAIL therapy reduced the tumor volume and downregulated the expression levels of STAMBPL1 and c-FLIP in an in vivo xenograft model. 

Previous studies have discovered that curcumin, a crucial active molecule present in *Zingiber officinale*, inhibits tumor cell apoptosis by targeting thioredoxin reductase (TrxR) [18]. TrxR is a significant target for anticancer drugs [19,20]. Zhang et al. have discovered that 6-Shogaol (6-S), an analog of curcumin, selectively inhibits purified TrxR1 activity by targeting selenocysteine residues [21]. The systematic revelation of 6-S as a TrxR inhibitor in cell models provides an experimental basis for developing 6-S as an anticancer candidate by promoting oxidative-stress-mediated HeLa cell apoptosis.

Machine learning and AI play a crucial role in the recognition and identification of naturally occurring compounds [22]. Therefore, it is imperative to construct and apply new machine learning and AI methods. Lee and Chiang’s research group proposes two new machine learning-based frameworks: StackTHPred and GRDF. StackTHPred is applied to the recognition of tumor-homing peptides (THPs), which specifically bind and accumulate in tumor tissue [23]. The framework uses effective feature selection algorithms and three tree-based machine learning algorithms, surpassing existing THP prediction methods. On the other hand, GRDF combines deep graph representation and deep forest architecture to identify anticancer peptides (ACPs) [24]. The accurate prediction of ACPs is crucial for discovering and designing new cancer treatment methods, and the effectiveness of GRDF in identifying ACPs may successfully promote the accurate prediction of ACPs. Hence, StackTHPred and GRDF are useful in exploring and identifying THP and ACP, respectively, which can aid in the development of innovative cancer therapies.

Chirality plays a vital role in the design, discovery, and development of new drugs [25]. The chirality of natural anticancer products and their analogs is crucial for their effectiveness. Each enantiomer may display different behaviors regarding pharmacodynamics, pharmacokinetics, and toxicity [25]. Habala et al. summarize the most recent examples of natural anticancer chiral compounds and their analogs with diverse structures, as well as their varied mechanisms of anticancer effects [26]. The authors also discuss the latest research on the differences in biological activity between the single enantiomers of anticancer agents and their racemic mixtures. Understanding the stereochemistry of anticancer compounds helps us to understand some of the key processes of their toxicity to cancer cells and provides a reasonable basis for designing new anticancer drugs.

This Special Issue has not received papers that focus primarily on clinical trials or the clinical conversions of natural drugs. While drugs like paclitaxel and camptothecin are currently utilized in clinical practice, important natural-source anticancer drugs have yet to undergo clinical trials (https://clinicaltrials.gov (accessed on 15 October 2023). Thus, it is imperative to acknowledge the therapeutic potential that natural anticancer molecules possess.
